# A leucine-rich diet modulates the mTOR cell signalling pathway in the gastrocnemius muscle under different Walker-256 tumour growth conditions

**DOI:** 10.1186/s12885-019-5448-0

**Published:** 2019-04-11

**Authors:** Bread Cruz, André Oliveira, Gislaine Ventrucci, Maria Cristina Cintra Gomes-Marcondes

**Affiliations:** 1 Department of Structural and Functional Biology, Institute of Biology, University of Campinas, UNICAMP, CP 6109, 13083862, Campinas, São Paulo, Brazil; 2 Faculty of Medical Science and Health, Pontifical Catholic University of São Paulo, 13030-095 - Sorocaba, São Paulo, Brazil

**Keywords:** Cancer cachexia, Leucine, mTOR cell signalling pathway, Eukaryotic Initialisation factors; Walker-256 tumour

## Abstract

**Background:**

The exact signalling mechanism of the mTOR complex remains a subject of constant debate, even with some evidence that amino acids participate in the same pathway as used for insulin signalling during protein synthesis. Therefore, this work conducted further study of the actions of amino acids, especially leucine, in vivo*,* in an experimental model of cachexia. We analysed the effects of a leucine-rich diet on the signalling pathway of protein synthesis in muscle during a tumour growth time-course.

**Methods:**

Wistar rats were distributed into groups based on Walker-256 tumour implant and subjected to a leucine-rich diet and euthanised at three different time points following tumour development (the 7th, 14th and 21st day). We assessed the mTOR pathway key-proteins in gastrocnemius muscle, such as RAG-A-GTPase, ERK/MAP4K3, PKB/Akt, mTOR, p70S6K1, Jnk, IRS-1, STAT3, and STAT6 comparing among the experimental groups. Serum WF (proteolysis-induced factor like from Walker-256 tumour) and muscle protein synthesis and degradation were assessed.

**Results:**

The tumour-bearing group had increased serum WF content, and the skeletal-muscle showed a reduction in IRS-1 and RAG activation, increased PKB/Akt and Erk/MAP4K3 on the 21st day, and maintenance of p70S6K1, associated with increases in muscle STAT-3 and STAT-6 levels in these tumour-bearing rats.

**Conclusion:**

Meanwhile, the leucine-rich diet modulated key steps of the mTOR pathway by triggering the increased activation of RAG and mTOR and maintaining JNK, STAT-3 and STAT-6 levels in muscle, leading to an increased muscle protein synthesis, reducing the degradation during tumour evolution in a host, minimising the cancer-induced damages in the cachectic state.

## Background

Branched-chain amino acids, especially leucine, play a prominent role in the regulation of protein metabolism. Both in vivo and in vitro studies corroborate the fact that a diet supplemented with leucine stimulates protein synthesis, particularly in skeletal muscle [1–4], and may even reverse protein catabolism [5–7].

Cellular signalling in the setting of protein synthesis involves the regulation of protein turnover in skeletal muscle, a complex process that generally involves an interaction between gene transcription and protein translation and degradation. The stimulus and signal of this process each come from outside the cell; the primary stimulating agents are anabolic hormones, such as insulin, growth factors, glucose and nutrients, such as amino acids, especially leucine [8–10].

The mammalian protein kinase target of rapamycin, mTOR, plays a central role in the development of skeletal muscle stimulation through growth by nutrition. mTOR acts as a sort of strategic centre from which various cellular processes are regulated [11, 12]. This protein kinase is composed of the following two independently adjustable complexes: mTOR Complex 1 (mTORC1) and mTOR Complex 2 (mTORC2), where only mTORC1 is involved in cell signalling stimulated by nutrition, as focused here mainly in muscle cells, especially because further studies about this pathway in the cachectic host are necessary.

Leucine is the primary amino acid that activates protein synthesis in skeletal muscle [8, 9]. Some evidence suggests that amino acids use the same pathway as that used for insulin signalling, although the exact mechanism by which signalling in the mTOR complex occurs remains a subject of constant debate [13]. Considerable evidence regarding the mechanism by which amino acids act on mTOR pathway has been acquired from in vitro studies using cell culture techniques. This information indicates the need for further studies on the action of amino acids in vivo*,* the primary purpose of our research. Therefore, the primary purposes of our research include investigating the time-course of tumour growth under a leucine-rich diet and the effects of this diet on the activation of upstream and down-stream proteins involved in protein synthesis in the gastrocnemius muscle of Walker-tumour-bearing rats.

## Methods

### Diets

The semi-purified isocaloric diets were a normal-protein diet (C), containing 18% protein [14], and leucine (L), containing 18% protein plus 3% L-leucine. Both diets contained approximately 70% carbohydrate (sucrose, dextrin and starch), 7% fat (soybean oil) and 5% fibre (purified micro-cellulose). Vitamins and mineral mix, as well as cystine and choline, supplemented the diets. The control diet contained 1.6% L-leucine, and the leucine-rich diet contained 4.6% L-leucine, according to previous experimental studies [15].

### Animal

Females Wistar rats (*n* = 72 animals; 90-days-old, weighing 180–200 g) were obtained from the Centre/UNICAMP animal facilities (CEMIB/State University of Campinas, Brazil), and received food and water ad libitum under light-dark cycles (12/12 h each) and constant temperature (22 + 2 °C) and humidity (50–60%). The animals were distributed into 12 groups based on whether they were implanted with Walker-256 tumour cells (1 × 10^6^ viable cells, counted by trypan blue exclusion), their periods of tumour development analysis (7, 14 and 21 days after implantation), and whether they received leucine-rich diet supplementation. Each group contained a minimum of six animals as follows: 1. Control rats (7C; 14C and 21C) subjected to a normoproteic diet and euthanised on the 7th, 14th and 21st days of the experiment; 2. Leucine rats (7 L, 14 L and 21 L) subjected to leucine-rich diet and euthanised on the 7th, 14th and 21st days of the experiment; 3. Tumour-bearing rats (7 W, 14 W and 21 W) subjected to a normoproteic diet, implanted with Walker 256 carcinosarcoma cells and euthanised on the 7th, 14th and 21st days following tumour development; 4. Leucine tumour-bearing rats (7WL, 14WL and 21WL) subjected to leucine-rich diet, implanted with Walker 256 carcinosarcoma cells and euthanised on the 7th, 14th and 21st days after tumour development.

The rats remained in collective cages throughout the experimental period, and each group of animals was euthanised by decapitation after 7, 14 or 21 days of tumour inoculation in order to evaluate and establish trends regarding changes in muscle profiles of cell signalling processes and the effects of these changes on protein synthesis. The gastrocnemius muscle was dissected; portions were weighed, quickly frozen in liquid nitrogen and subsequently stored in a bio-freezer for further biochemical and molecular analyses. The general UKCCCR [16] guidelines for animal welfare were followed, and the Institutional Committee for Ethics in Animal Research approved the study protocol (CEEA/IB/UNICAMP, protocol number #2418–1).

### Multiplex analysis

The serum insulin was performed using an immunoassay kit (multiplex kit, Millipore, USA) according to the manufacturer’s instructions and expressed as pM, using a standard provided by the manufacturer with a range of 1 to 500 pM, and fluorescent flow cytometry with Luminex equipment (Millipore, USA).

The gastrocnemius muscle samples were homogenised in a buffer sample (Milliplex MAP Cell Signalling Buffer – Millipore, USA) according to the manufacturer’s instructions, and analyses of the phosphorylated signalling proteins IRS-1, Stat-3, Stat-6, Jnk, ERK/MAPK, Akt-PKB, p70S6K and mTOR were completed using multiplex kits (Multiplex MAP Cells Signalling Millipore, USA), according to the manufacturer’s instructions. The fluorescence of coupling beads with capture antibodies specific for each protein of interest was measured using the Luminex®200™ system (Luminex Corporation, TX, USA). The analyses were carried out using xPonent® 3.1 Software (Luminex Corporation, TX, USA) and the Luminex®200™ system. The protein phosphorylation are as follows: p-IRS-1^panTyr^, p-Akt/PKB^Thr308^, p-ERK/MAPK ^Thr185/Tyr187^, p-mTOR^Ser2448^, p-70S6K^Thr412^, p-STAT3^Ser727^, p-STAT6^Tyr641,^ p-Jnk ^Thr/Tyr185^, as provided by the manufacturer, and the Millipore cell signalling kits tested for each antibody, using the IP Western assay; the HeLa cell lysate was used as a positive control (provided by the Milliplex map kit detection by Millipore).

### Western blot

Serum samples were also resolved to analyse the proteolysis-induced factor-like Walker Factor (WF) as previously described by Yano and colleagues [17]. The primary anti-WF antibody was produced at the Nutritional Medicine Lab, School of Life and Health Sciences, Aston University, Birmingham, UK, and gentle donated by Dr. Michael J. Tisdale for use in our experiments.

The gastrocnemius muscles were homogenised with homogenisation buffer (Tris Base 100 mM, Na4P2O7 10 mM, FNa 100 mM, Na3VO4 1 mM, EDTA 10 mM, PMSF 2 mM, Aprotinin 0,1 mg/mL, Triton X-100 1%, pH 7.4), centrifuged, and divided into aliquots for a total protein analysis [18]. We also verified the muscle expression of RAS-related GTPase (RAG-A) (Affinity, USA, and diluted 1:1500). Either 2.5 μg of muscle homogenate protein or serum total protein were resolved in the SDS-acrylamide gel (12%) and transferred to 0.45 μm nitrocellulose membrane. The protein expression of RAG-A in muscle and WF in serum was assessed using secondary anti-rabbit antibodies. Each protein expression level was corrected against a GAPDH control load. The reaction was determined via chemiluminescence using an ECL reagent (Amersham). Densitometry analysis of the protein bands was performed using Image Capture (Amersham) and analysed using Gel-Pro II software.

### Protein synthesis and protein degradation assays

The protein synthesis was assayed in right gastrocnemius muscles, as described by Ventrucci et al. [19]. The muscles were weighed and placed in Krebs-Henseleit bicarbonate (KHB) buffer (110 mM NaCl; 25 mM NaHCO_3_; 3.4 mM KCl; 1 mM CaCl_2_; 1 mM MgSO_4_ and 1 mM KH_2_PO_4_, pH 7.4) supplemented with 5.5 mM glucose and 0.01% (*w*/*v*) albumin. The muscles were pre-incubated for 30 min at 37 °C with continuous gassing (95% O_2_–5% CO_2_). After this, new KHB buffer supplemented with 5 μCi/ml of L-(^3^H) phenylalanine (Amersham Life Science, NY, USA) was added, and the incubation continued for further 2 h. At the end of this period, the muscles were homogenised in trichloroacetic acid (TCA 10%, 1:3 *w*/*v*), centrifuged at 10.000×g and measured the total protein [18] and quantity the radioactivity based on liquid scintillation of β emissions. The rate of protein synthesis was calculated by the amount of radioactive phenylalanine incorporated in the 2-h period and was expressed as nmols (^3^H)-Phe per mg of muscle protein [20].

Protein degradation was assessed using the left gastrocnemius muscles after rapidly excised, were placed in RPMI 1640 medium and pre-incubated for 30 min under the same conditions of temperature and gas as described above. After the initial incubation, the solutions were replaced by KHB supplemented with cycloheximide (130 μg.mL^− 1^) followed further 2 h incubation. The rate of muscle protein degradation was determined as nmol of tyrosine released per mg of muscle protein/ hour, based on a fluorimetric assay described by Waalkes & Udenfriend [21].

The leucine and KIC incorporations into gastrocnemius muscle protein were assessed in other new muscles samples from new similar experiments where the groups were euthanised on 21st day of tumour evolution. Leucine incorporation was assayed in right gastrocnemius muscles, after weighing and placing the muscles in KHB buffer supplemented with 1.26 mmol/L leucine. The KIC incorporation into muscle protein was assayed in left gastrocnemius muscles which were weight and placed in KHB buffer supplemented with 0.26 mmol/L leucine and 1.0 mmol/L KIC. The right and left muscles were pre-incubated, separately, for 30 min at 37 °C with continuous gassing (95%O_2−_ 5%CO_2_). After this interval, new KHB buffer supplemented with (^3^H)-leucine (10 μCi.L^− 1^) or (^14^C) KIC (50 μCi.L^− 1^) (Amersham Life Science, NY, USA) were added as a tracer, and the incubation continued for further 2 h. At the end of this period, the muscles were processed as the same procedures described above, homogenising in trichloroacetic acid, and assessed to measure the total protein [18] and quantity the radioactivity based on liquid scintillation of β emissions. The rates were calculated by the amount of radioactive of leucine and KIC incorporated in 2-h period and were expressed as nmols (^3^H)-Leu and (^14^C)-KIC per mg of muscle protein [20].

### Statistical analyses

The results were expressed as the mean ± standard deviation. Two-way ANOVA followed by Fisher’s LSD multiple comparison test was used to compare the experimental groups with the control group. A value of *P* < 0.05 was considered significant [22].

## Results

Our results showed the consequences of tumour evolution in muscle protein signalling during a time-course of Walker-256 tumour growth, and as present here, we showed that leucine participated in signalling pathways in parallel to insulin stimulation, modulating the Akt-PKB pathway, and also mTOR via RAG GTPases.

### Leucine-rich diet minimised the changes in insulin and proteolysis-induced factor like – Walker factor – serum content

**Fig. 1:**
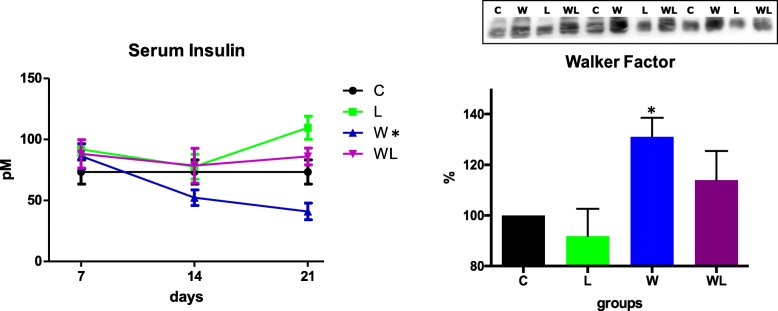
A profile of serum insulin (pM), a proteolysis-induced factor (Walker factor, WF, % compared to the control group). The animals were euthanised at different times during tumour development to assess the profile of serum insulin including the 7th, 14th, and 21st days of the experiment. The Walker Factor was assessed at 21st day of the experiment. Legend: C, control group; W, Walker tumour-bearing group; L, rats fed a leucine-rich diet; WL, tumour-bearing rats fed a leucine-rich diet. Graphics represent mean ± standard deviation, and statistical significance is presented in Table 1 after two-way Anova analysis, followed by the Fisher’s LSD multiple comparison test; *P* < 0.05

We observed an increased content of Walker factor in tumour-bearing rats at the 21st day (pre-agonic state) (Fig. 1), and despite having a tumour, the leucine tumour-bearing group (WL) showed a 14% lower content compared to W group (Fig. 1). The serum insulin decreased in the tumour-bearing group (W), starting on the 14th day, and became more prominent following the 21st day of the experiment (Fig. 1 and Table 1). On the other hand, the leucine tumour-bearing group (WL) maintained the serum insulin levels compared with the control group, being different from the W group (Fig. 1 and Table 1).

**Table 1:** A statistical analysis of serum content and the muscle key proteins of a protein synthesis pathway

	7th day	14th day	21st day
Serum Insulin profile	n.s.	*W < C	*W < C *L > C *W < WL
Serum Walker Factor	n.d.	n.d.	*W > C *W > L *W > WL; *P* = 0.0517
Phospho IRS-1/total IRS-1 ratio	*W < C	*W < C; *P* = 0.056	n.s.
Phospho AKT-PKB	n.s.	*WL < L	*W > C *W > L *WL < W
Phospho ERK-MAPK	n.s.	n.s.	*W > C *W > L
Phospho mTOR	n.s.	n.s.	*WL > C *WL > L *WL > W
Phospho p70S6K	n.s.	n.s.	n.s.
pmTOR/p70S6K-1 ratio	*L > C *WL < C	n.s.	*W < C *WL > C,L *WL > W
Phospho RAG-A	*L > C *WL > C *WL > L	*L > C *W > C *WL > C	*L > C *WL > C *W < L *WL > W
Phospho JNK	*W > WL	n.s.	n.s.
Phospho STAT3	n.s.	n.s.	*W > C
Phospho STAT6	n.s.	*W > C	*W > C *W > L
Phenylalanine Incorporation	n.d.	n.d.	*C > W *L > WL *WL > W
Leucine Incorporation	n.d.	n.d.	*W < C; *p* = 0,058
KIC Incorporation	n.d.	n.d.	*C > W *WL > W
Tyrosine release	n.d.	n.d.	*W > C *WL > L *W > WL
Total protein net	n.d.	n.d.	*C > W *L > WL *WL > W

Legend: C, control group; W, Walker tumour-bearing group; L, rats fed a leucine-rich diet; WL, tumour-bearing rats fed a leucine-rich diet. Two Way ANOVA analysis followed by the Fisher’s LSD multiple-comparison test (*P* < 0.05 values). * The mean difference of 0.05 is statistically significant. n.s. = non-significant. n.d. = non-determined. For details, see the Materials and Methods and Results sections

### Leucine-rich diet increased the phosphorylation of RAG-A and increased the pmTOR/pp70S6K1 ratio in muscle

**Fig. 2:**
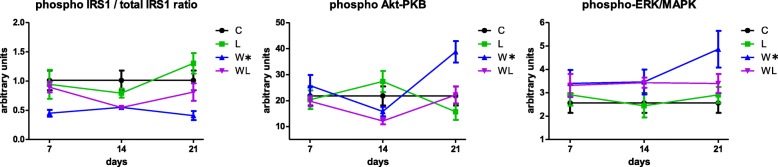
Key-proteins related to the mTOR pathway: phosphorylated Akt-PKB, Erk/MAPK, and IRS-1 in the gastrocnemius muscle under the influence of Walker-tumour development and nutritional supplementation with leucine. The minimum number of animals per group was 6. The animals were euthanised at different times during tumour development, including the 7th, 14th, and 21st days of the experiment. Legend: C, control group; W, Walker tumour-bearing group; L, rats fed a leucine-rich diet; WL, tumour-bearing rats fed a leucine-rich diet. Graphics represent mean ± standard deviation. Statistical significance is presented in Table 1 after two-way Anova analysis, followed by the Fisher’s LSD multiple comparison test; *P* < 0.05

**Fig. 3:**
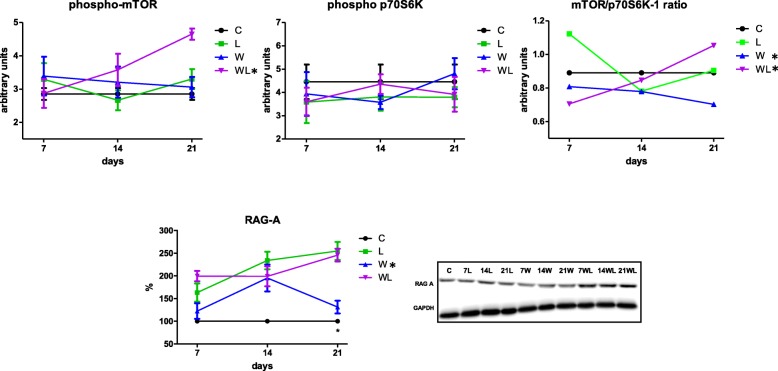
mTOR, p70S6K-1, mTOR/p70S6K ratio and RAG-A (arbitrary units). The animals were euthanised at different times during tumour development, including the 7th, 14th, and 21st days of the experiment. Legend: C, control group; W, Walker tumour-bearing group; L, rats fed a leucine-rich diet; WL, tumour-bearing rats fed a leucine-rich diet. Graphics represent mean ± standard deviation. Statistical significance is presented in Table 1 after two-way Anova analysis, followed by the Fisher’s LSD multiple comparison test; *P* < 0.05

In order to investigate the effects of tumour development on muscle protein signalling, we analysed several key proteins of mTOR pathway obtained from rats at 7, 14 and 21 days following tumour development, analysing the proteogenesis cell signalling via. We verified that the tumour development, as a time-course analysis, the phospho IRS1/total-IRS1 ratio was decreased in W group at 7th and 14th day after tumour implant in comparison to control group. In the opposite way, the phospho AKT-PKB and phosphor ERK-MAPK increased in muscle of tumour-bearing group (W) at the 21st day, compared to the control group (Fig. 2; Table 1). Although the phospho-mTOR and phosphor-p70S6K1 had a similar expression in W group compared to C group along the experiment, but decreased phosphor-mTOR/pp7-S6K1 ratio at the 21st day (Fig. 3, Table 1).

On the other hand, analysing the tumour-bearing animals subjected to leucine-rich diet, the Fig. 2 showed that the WL group exhibited similar of IRS-1 activity levels as those of the C group, which were slightly higher than those of the W group on the 21st day of the experiment (WL showed 1.9-times higher than W; *P* = 0.0628). There are some evidence that leucine uses parallel stimulation via mTOR and does not directly participate in the modulation of Akt-PKB or activation of Erk/MAPK, therefore, we verified similar values of muscle phospho AKT-PKB and phosphor ERK-MAPK in WL group when compared to control and leucine groups (Fig. 2 and Table 1). The analysis of the concentration of phospho-mTOR in the gastrocnemius muscle revealed a significant increase in this concentration in the WL group especially at the 21st day of the experiment compared with the other groups (statistical significance noted on only the 21st day; Fig. 3; Table 1). The WL group had similar values of phospho p70S6K1, but the muscle phospho-mTOR/pp70S6K1 ratio increased on the 21st day in this group WL compared to W group (WL > 1.7-times; *P* = 0.0217). This difference was more evident on the 21st day of the experiment, as the groups that received leucine supplementation demonstrated activation at this point of the pathway, which could be evidenced by enhanced values of RAG-A expression especially in both groups subjected to leucine-rich diet. Despite having a tumour, the WL group had an increase in RAG-A 1.9-times when compared to W group (*P* = 0.004), which likely indicated that the stimulus that acted on the mTOR complex mostly resulted from the activation of RAG-A GTPases (Fig. 3 and Table 1).

### Leucine-rich diet modulated the downstream component of mTOR pathway

**Fig. 4:**
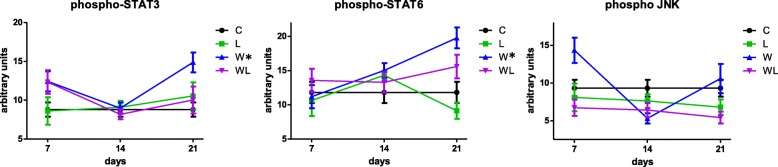
A profile of phosphorylated JNK, STAT3 and STAT6 in the gastrocnemius muscle under the influence of Walker-tumour development and nutritional supplementation with leucine. The minimum number of animals per group was 6. The animals were euthanised at different times during tumour development, including 7th, 14th, and 21st day of the experiment. Legend: C, control group; W, Walker tumour-bearing group; L, rats fed a leucine-rich diet; WL, tumour-bearing rats fed a leucine-rich diet. Graphics represent mean ± standard deviation. Statistical significance is presented in Table 1 after two-way Anova analysis, followed by the Fisher’s LSD multiple comparison test; *P* < 0.05

In order to answer the question regarding the modulatory effect of leucine supplementation, we analysed the STAT3 protein, a downstream component of mTOR complex 1 (mTORC1); Fig. 4 shows the profile of phospho-STAT3 among the groups, and only on day 21 showed difference between W and C groups (W > C; *P* = 0.0174) and also against the WL (W > WL 1.5-times; *P* = 0.0382) (Fig. 4; Table 1). Adding to this analysis, the levels of phospho-STAT6 were not significantly different during the first two periods of the analysis (7 and 14 days) among the groups, except comparing W group with C at the 14th day (W > C; *P* = 0.013) (Fig. 4). On day 21, phospho-STAT6 showed differences in tumour-bearing groups when compared to their respective groups without a tumour (phospho-STAT6 was higher in W vs C; *P* = 0.0287, and was increased in WL compared to L group; *P* = 0.0087) (Fig. 2 and Table 1). The stress-activated c-Jun amino-terminal kinase (JNK), which plays a pivotal role in several metabolic conditions, increased only in the skeletal muscle of the W group compared with other groups on the 7th day and the 21st day experiment compared with the C and WL groups on the same days of sacrifice (day 7 and day 21) (Fig. 4, Table 1).

### Protein synthesis is increased in tumour-bearing group subjected to leucine-rich diet

**Fig. 5:**
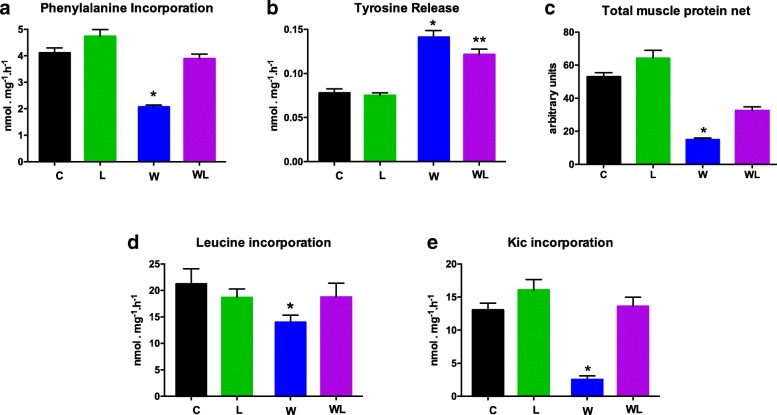
Effects of a leucine-rich diet in gastrocnemius skeletal muscle from tumour-bearing rats. **a** Phenylalanine incorporation, **b** Tyrosine release, **c** Total muscle protein net (represented by phenylalanine/tyrosine ratio), **d** Leucine incorporation, **e** KIC incorporation into muscle protein in different groups. For details, see Methods. Abbreviations: C, control; L, leucine-rich diet group; W, tumour-bearing rats; WL, tumour-bearing rats fed a leucine-rich diet. Graphics represent mean ± standard deviation. Statistical significance is presented in Table 1 after two-way Anova analysis, followed by the Fisher’s LSD multiple comparison test; *P* < 0.05

In order to investigate the positive effects of leucine-rich diet, we accessed the protein synthesis and degradation in the muscle of all groups. The phenylalanine incorporation into gastrocnemius muscle protein is shown in Fig. 5a. Tumour growth reduced phenylalanine incorporation in both tumour-bearing groups, W and WL (Fig. 5a, Table 1), compared to C and L groups, but the leucine tumour-bearing group (WL) showed a 1.87-fold higher incorporation of phenylalanine (3.88 ± 1.79 nmol (^3^H)-Phe /mg/h) than W group (W = 2.07 ± 0.06 nmol (^3^H)-Phe /mg/h) (Fig. 5a, Table 1).

With the same point of view, tumour growth reduced leucine incorporation into muscle only in W group. However, the leucine incorporation in the tumour-bearing group fed the leucine-rich diet (WL) was not significantly different but showed an increased value (WL = 18.77 ± 2.57 nmol [^3^H]-Leu/mg/h) when compared to W group (W = 14.00 ± 1.31 nmol [^3^H]-Leu/mg/h) (Fig. 5d, Table 1).

KIC incorporation indicates protein synthesis, stimulated by indirect effect of leucine, which could be affected by tumour growth, as we noted here where the W group had a decrease in muscle KIC incorporation (Fig. 5e, Table 1), but was 5.4-times higher in WL than in W group (W = 2.50 ± 0.58 versus WL = 13.58 ± 1.39 nmol [^14^C]-KIC /mg/h).

### Protein degradation is decreased in tumour-bearing group with leucine-rich diet

In order to analyse the tumour wasting effect, we analysed the muscle protein degradation by tyrosine released which was increased in both tumour-bearing groups, W and WL, compared to C and L groups (Fig. 5b). These data reflected directly on total protein net (muscle’s phenylalanine incorporation and tyrosine release ratio) (Fig. 5c), which was notably reduced (~ 53%) in W group compared to the other WL groups.

## Discussion

The mTOR complex integrates various signalling pathways that are modulated by different stimuli, including insulin, hypoxia, energy stress and nutrients [23]; the last group stands out, as amino acids may serve as stimulants, particularly leucine. Recent studies have shown that leucine participates in signalling pathways in parallel to those used by other stimuli [11, 24]. Therefore, although stimulation by insulin modulates the Akt-PKB pathway, leucine modulates mTOR via RAG GTPases [25, 26].

The present work contributed to deepening further the results obtained in previous studies [27]. Therefore, the present work analysed components of the mTOR cell signalling pathway under the influence of leucine, in the setting of cachexia; as mentioned above, this is the first time that the effects of tumour development on muscle protein synthesis were analysed during the course of an experiment. Furthermore, the analysis of these proteins at different stages of tumour development provided us with more detailed insights into the time course of activation or modulation as a result of nutritional supplementation with leucine, which may be important in the management of co-adjuvant therapy in patients with cancer.

**Fig. 6:**
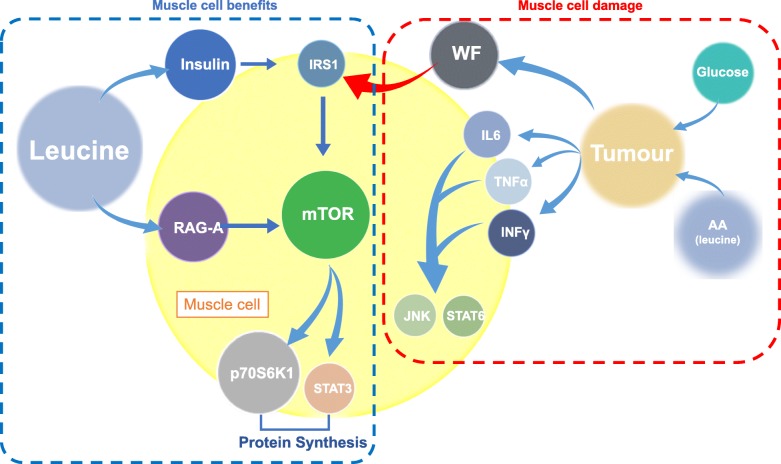
A scheme showing variations of key phosphorylated protein concentrations in the gastrocnemius muscle under the influence of tumour development on different days of the experimental protocol (7th, 14th and 21st day), and under the influence of nutritional supplementation with leucine

Briefly, the Walker-256 tumour as an experimental model of cachexia shows that its development increases the muscle protein degradation by increasing the proteolysis-induction factor-like – Walker-Factor - (WF) levels, but also decreases the muscle protein synthesis by reducing the insulin level, which was associated with lower activation of IRS-1 in W group, especially after the 14th and 21st day after implant. In parallel, we verified increases in the levels of phosphorylated Akt-PKB and ERK/MAPK only at 21 days of tumour growth. Although, these high levels of Akt did not lead to activation of mTOR via in muscle of these tumour-bearing hosts, which clearly showed that the mTOR pathway was compromised likely starting by the 14th day of tumour development. On the other hand, one of the downstream components of mTORC1, phospho-STAT3 had some variable profile with an expressive rise in the muscle of these tumour-bearing groups on day 21. Indeed, STAT3 mediates the expression of a variety of genes in response to cellular stimuli and therefore plays a key role in many cellular processes, including cell apoptosis and inflammatory processes [27, 28]. STAT3 promotes oncogenesis by being constitutively active in various pathways characterised by the activity of cytokines and growth factors, including interleukins IL-6, as previously noted in our study [29]. Therefore, we hypothesised that the presence of tumour cells impaired the function of the mTOR pathway, which paralleled the effects attributed to increased phosphor-STAT3 levels in muscle cells. Additionally, the levels of phospho-STAT6 showed a rising level, which was evidenced on the 21st day of tumour development (W group). This protein plays a central role in IL-4-mediated biological responses during the inflammatory process [30]. It induces the expression of BCL2L1/BCL-X, which is responsible for the anti-apoptotic activity of IL-4 [31]. Then, we likely suggested that on 21st day, the tumour-bearing group (21 W) was subjected to more intense effects of the inflammatory process, higher interleukin IL-4 are associated with increased levels of IL-6, TNF and INF in the W group [29]. In addition, the stress-activated c-Jun amino-terminal kinase (JNK), which plays a pivotal role in several metabolic conditions, increased on 21st day in the skeletal muscle of the W group. This protein participates in the cell-signalling pathway associated with stress and inflammatory processes [32]. Tumour-bearing hosts, particularly those of the animals in the W group, are in a process characterised by inflammation and stress in the setting of cachexia [29, 33, 34]. All these points closely connected with the inflammatory process associated with the higher Walker-Factor levels in these tumour-bearing rats inducing a low activity of mTOR pathway associated with an increase on inflammatory pathways (Fig. 6).

In order to investigate some of the benefits of leucine-rich diet over the damage effects of tumour development on muscle protein, here we analysed the profile of these same key proteins of mTOR signalling pathway, including phosphorylated proteins, from up-stream and down-stream steps, which provided some interesting point how the leucine-rich diet could modulate the tumour-induced damages in this experimental model of cachexia.

In fact, the leucine-rich diet clearly demonstrated that could ensure maintenance of serum insulin levels throughout the course of tumour development, improving the muscle protein synthesis. Indeed, as presented here, Petruzzelli et al. [35] observed a reduction in serum insulin levels, particularly reduced insulin secretion, after glucose stimulation, in islet cells isolated from Walker 256 tumour-bearing rats. Insulin is an important hormone and plays a prominent role in the physiologic changes that occur in the setting of cachexia. For this reason, we hypothesised that the WL group would most likely benefit from nutritional supplementation, as muscle protein was less wasted in W group (phenylalanine incorporation and total protein net). It may be noted that the administration of leucine notably modulated insulin and also the proteolysis-induction factor-like (WF) levels, which led to an improvement effect on the mTOR cell signalling. The presence of leucine causing increases in serum insulin content [11, 36, 37] and in part modulating the WF levels, likely suggests the effect leading to increased IRS-1 activation. On the other hand, we confirmed that the leucine-rich diet did not appear to have affected signalling, by unchanged levels of phosphorylated Akt-PKB or ERK/MAPK, during the course of tumour development. A recent study demonstrated that leucine uses parallel stimulation via mTOR and does not directly participate in the modulation of Akt-PKB [13].

Indeed, the analysis of the concentration of phosphor-mTOR in the gastrocnemius muscle revealed an increase in this concentration in the tumour-bearing group that received a leucine-rich diet between the 14th and the 21st day of the experiment compared with the control group and also with the non-supplemented tumour-bearing group. Interestingly, leucine has been described by some researchers as an inhibitor of protein degradation and a stimulator of protein synthesis in cachectic hosts [5, 38]. In this present study, we observed results that are consistent with those noted by other researchers, as we found that leucine most likely stimulates protein synthesis through the mTOR signalling pathway (as noted in the WL group), particularly over longer time, such as 14 or 21 days of tumour development. Indeed, the higher phospho-mTOR concentrations are associated with higher serum insulin concentration, as verified by other studies with leucine-rich diet [29, 39, 40]. This fact was also confirmed by the amino-acid-signalling to mTORC1, which is mediated by Rag GTPases [13]; additionally, we observed that leucine led to a higher level with the difference more evident on the 21st day of the experiment in tumour-bearing group WL (Fig. 3 and Table 1). The leucine supplementation clearly demonstrated activation at this point of the pathway, which indicates that the stimulus that acted on the mTOR complex most likely resulted from the activation of RAG GTPases (Fig. 3). Amino acids signal the mechanistic and mammalian TOR complex 1 (mTORC1) via the RAS-related GTP-binding protein (RAG) family of small GTPases ([12, 13, 26]. The RAGs are assembled into heterodimers containing RAG-A (as verified in this study), a process stimulated by amino acids, particularly leucine [12]. In spite of the evidence that mTOR was not permanently increased in the L group, suggesting an adaptive process that reached a steady state following 3 weeks of leucine-rich dietary supplementation, the WL tumour-bearing group showed that RAG-A facilitated the stimulation of the mTOR pathway, confirming the muscle response in the WL group that was noted in previous studies [7, 10, 39, 40]. Meanwhile, in order to answer the question regarding the modulatory effect of leucine supplementation, we analysed the downstream component of mTOR complex 1 (mTORC1), the phospho p70S6K protein demonstrated only limited variation among the groups, which indicated that this portion of the mTOR pathway was only minimally affected by leucine supplementation. As mentioned above, we hypothesised that the adaptive process involving leucine supplementation might stimulate the mTOR pathway; thus, the analysis of mTOR/p70S6K-1 ratio made it possible to determine that a stronger stimulus occurred in the L group on the 7th day and continued as the experiment progressed (14th and 21st day). Analysing the tumour effect, the mTOR/p70S6K-1 stimulus that occurred in WL group during the experiment differed from that observed in the W group, especially after the 14th day, which was perfectly fitted to the raising of phospho-mTOR and RAG-A expression, suggesting the positive effect of leucine supplementation.

Considering the activation of downstream mTOR pathway, the maintenance of STAT3 phosphorylation occurred in WL group showed the leucine influence, since the group without tumour cells or bearing a tumour had constant levels of phospho-STAT3 over the three study days compared with the C group. Indeed, STAT3 mediates the expression of a variety of genes in response to cellular stimuli, in this case being modulated by leucine effects. We suggest that the STAT3 could be likely activated in various pathways characterised by the activity of cytokines and growth factors, including interleukins IL-6, as previously noted in your study [29], but under leucine supplementation, this downstream protein could be reduced benefiting the skeletal muscle in these tumour-bearing animals (Fig. 4). Constitutive STAT3 activation is associated with various human cancers and often suggestive of a poor prognosis, mostly related to inflammatory processes [27]. However, leucine supplementation interfered with this process, as muscle STAT3 activation was similar to that noted in the control groups (C and L; Fig. 4). In this same view, the raises of phospho-STAT6 and JNK in muscles of tumour-bearing rats leads us to the thought that the muscle catabolic process was directly related to the increase of pro-inflammatory cytokines from day 14th of tumour development, as previously shown by Cruz and colleagues [29]; although the leucine-rich diet also contributed to reducing this tumour-induced damage over skeletal muscle decreasing these proteins especially after day 14 of the experiment (Fig. 4). We hypothesised that on 21st day, the tumour-bearing group was subjected to more intense effects of the inflammatory process [29]. This change was smaller in the tumour-bearing rats subjected to leucine supplementation. The stress-activated c-Jun amino-terminal kinase (JNK) participates in the cell-signalling pathway associated with stress and inflammatory processes, which in particularly those of the animals in the W group are in inflammation and stress processes in the setting of cachexia, particularly when the Walker-tumour cell burden exceeds 10% of an animal’s body weight (in this study, the tumour/body weight ratio reached more than 12%, [29]), as noted previously by several researchers [34, 41, 42]. As noted in our previous studies, leucine stimulates protein synthesis [10, 39, 40]; therefore, based on the results obtained in this study, it is possible that a component acting in parallel to the mTOR signalling pathway acts as a result of leucine stimulation, leading to increase in muscle protein synthesis, minimising the effects of the tumour cells as a result of decreasing WF levels.

After analyse those time-course experiments we choose the 21st day as a point of more pronounced effect of a tumour and leucine-rich diet on animals’ body. Therefore, radioactive experiments were performed only on the 21st day of tumour development.

Leucine stimulates muscle protein synthesis and modules the activity of various proteins involved in the control of mRNA translation [43]. Leucine can stimulate protein synthesis directly and/or via its metabolite, as α-ketoisocaproic acid (KIC). These findings are corroborated by the data on protein synthesis and degradation contained in Fig. 5. It can be noted that the presence of leucine stimulates protein synthesis through increased incorporation of phenylalanine and KIC (a ketoacid converted from Leucine). In contrast, the group fed a control diet and tumour-bearing presents lower protein synthesis and higher degradation rates. Since protein synthesis is reduced and protein degradation is increased throughout the body during cancer cachexia, the incorporation of amino acids, especially branched-chain amino acids, by muscle cells could supply important nutrients and influence protein synthesis. The lower incorporation of leucine into gastrocnemius muscle proteins in tumour-bearing rats indicated a decrease in protein synthesis that paralleled an increase in protein wasting [40, 44, 45]. As shown here, the leucine-rich diet reduced the protein catabolism and increased the protein synthesis in WL rats compared to W rats.

These finds clearly show the minimisation of tumour-induced damage effects when these tumour-bearing rats were subjected to leucine-rich diet. Leucine supplementation appeared to reduce the effects of stress in the host body, as well as effects of the inflammatory processes generated by the presence of the tumour cells improving the host’s responses and maintaining lean body mass (Fig. 6).

## Conclusions

This work demonstrates for the first time a time-course study with action of leucine anticipating and enhancing the parameters analysed in the setting of tumour growth; this involves the stimulation of protein synthesis, which results in reduced cachexia and occurs primarily in the experiment that achieves the best values on the 21st day following the start of the experiment. These results are consistent with those observed in previous studies performed by our research group. It was also noted that leucine stimulates various points of the mTOR pathway, including different anabolic hormones via, such as insulin. Further study is necessary in order to completely understand the effects of leucine on protein synthesis and protein degradation at the molecular level.

## Abbreviations

14 L: Leucine rats subjected to leucine-rich diet and euthanised on the 14th day of the experiment
14 W: Tumour-bearing rats subjected to a normoproteic diet, implanted with Walker 256 carcinosarcoma cells and euthanised on the 14th day following tumour development
14C: Control rats subjected to a normoproteic diet and euthanised on the 14th day of the experiment
14WL: Leucine tumour-bearing rats subjected to leucine-rich diet, implanted with Walker 256 carcinosarcoma cells and euthanised on the 14th day after tumour development
21 L: Leucine rats subjected to leucine-rich diet and euthanised on the 21st day of the experiment
21 W: Tumour-bearing rats subjected to a normoproteic diet, implanted with Walker 256 carcinosarcoma cells and euthanised on the 21st day following tumour development
21C: Control rats subjected to a normoproteic diet and euthanised on the 21st day of the experiment
21WL: Leucine tumour-bearing rats subjected to leucine-rich diet, implanted with Walker 256 carcinosarcoma cells and euthanised on the 21st day after tumour development
7 L: Leucine rats subjected to leucine-rich diet and euthanised on the 7th day of the experiment
7 W: Tumour-bearing rats subjected to a normoproteic diet, implanted with Walker 256 carcinosarcoma cells and euthanised on the 7th day following tumour development
7C: Control rats subjected to a normoproteic diet and euthanised on the 7th day of the experiment
7WL: Leucine tumour-bearing rats subjected to leucine-rich diet, implanted with Walker 256 carcinosarcoma cells and euthanised on the 7th day after tumour development
ERK/MAP4K3: Mitogen-activated protein kinase 3
IRS-1: Insulin receptor substrate 1
JNK: stress-activated c-Jun amino-terminal kinase
mTOR: mechanistic target of rapamycin
p70S6K1: Ribosomal protein S6 kinase beta-1
PKB/Akt: Protein kinase B
RAG-A-GTPase: Rag small GTPases heterodimer A
STAT3: Signal transducer and activator of transcription 3
STAT6: Signal transducer and activator of transcription 6
Walker-256 tumour: Wistar rats carcinosarcoma
WF: proteolysis-induced factor like from Walker-256 tumour
